# Correction: Medeiros-Ventura et al. The Impact of Phytase and Different Levels of Supplemental Amino Acid Complexed Minerals in Diets of Older Laying Hens. *Animals* 2023, *13*, 3709

**DOI:** 10.3390/ani14121829

**Published:** 2024-06-20

**Authors:** Waleska R. L. Medeiros-Ventura, Carlos B. V. Rabello, Marcos J. B. Santos, Mércia R. Barros, Rogério V. Silva Junior, Heraldo B. Oliveira, Fabiano S. Costa, Andresa G. Faria, Alba K. Fireman

**Affiliations:** 1 Animal Science Department, Universidade Federal Rural de Pernambuco, Rua Dom Manoel de Medeiros, s/n, Dois Irmãos, Recife 52171-900, Brazil; waleska_medeiros@hotmail.com (W.R.L.M.-V.); carlos.rabello@ufrpe.br (C.B.V.R.); rogerio-ventura@hotmail.com (R.V.S.J.); heraldoliveira1@gmail.com (H.B.O.); andresafaria2017@gmail.com (A.G.F.); 2 Veterinary Science Department, Universidade Federal Rural de Pernambuco, Rua Dom Manoel de Medeiros, s/n, Dois Irmãos, Recife 52171-900, Brazil; mercia.barros@ufrpe.br (M.R.B.); fabianosellos@hotmail.com (F.S.C.); 3 Zinpro Corporation, Eden Prairie, MN 55344, USA; afireman@zinpro.com

To allow the original publication to be better understood [1], the authors would like to make the following updates.

## Figure/Table Legend

Table 3

**In the original publication, there was a mistake in the legend for** “AACM-100 or AACM-EZ-100: 60, 70, 8, 40, 0.250 mg kg^−1^ of Zn, Mn, Cu, Fe, I, and Se, respectively; AACM-70 or AACM-EZ70: 42, 49, 6, 28, 0.175 mg kg^−1^ of Zn, Mn, Cu, Fe, I, and Se, respectively; AACM-40 or AACM-EZ-40: 24, 28, 3, 16, 0.100 mg kg^−1^ of Zn, Mn, Cu, Fe, I, and Se, respectively”. **should be replaced with** “AACM-100 or AACM-EZ-100: 60, 70, 8, 40, 1.0, and 0.250 mg kg^−1^ of Zn, Mn, Cu, Fe, I, and Se, respectively; AACM-70 or AACM-EZ70: 42, 49, 6, 28, 0.700, and 0.175 mg kg^−1^ of Zn, Mn, Cu, Fe, I, and Se, respectively; AACM-40 or AACM-EZ-40: 24, 28, 3, 16, 0.400, and 0.100 mg kg^−1^ of Zn, Mn, Cu, Fe, I, and Se, respectively”.

Table 4

**In the original publication, there was a mistake in the legend for** “AACM-100 or AACM-EZ-100: 60, 70, 8, 40, 0.250 mg kg^−1^ of Zn, Mn, Cu, Fe, I, and Se, respectively; AACM-70 or AACM-EZ-70: 42, 49, 6, 28, 0.175 mg kg^−1^ of Zn, Mn, Cu, Fe, I, and Se, respectively; AACM-40 or AACM-EZ-40: 24, 28, 3, 16, 0.10 mg kg^−1^ of Zn, Mn, Cu, Fe, I, and Se, respectively”. **should be replaced with** “AACM-100 or AACM-EZ-100: 60, 70, 8, 40, 1.0, and 0.250 mg kg^−1^ of Zn, Mn, Cu, Fe, I, and Se, respectively; AACM-70 or AACM-EZ70: 42, 49, 6, 28, 0.700, and 0.175 mg kg^−1^ of Zn, Mn, Cu, Fe, I, and Se, respectively; AACM-40 or AACM-EZ-40: 24, 28, 3, 16, 0.400, and 0.100 mg kg^−1^ of Zn, Mn, Cu, Fe, I, and Se, respectively”.

Table 5

**In the original publication, there was an error for** “AACM-100 or AACM-EZ-100: 60, 70, 8, 40, 0.250 mg kg^−1^ of Zn, Mn, Cu, Fe, I, and Se, respectively; AACM-70 or AACM-EZ-70: 42, 49, 6, 28, 0.175 mg kg^−1^ of Zn, Mn, Cu, Fe, I, and Se, respectively; AACM-40 or AACM-EZ-40: 24, 28, 3, 16, 0.100 mg kg^−1^ of Zn, Mn, Cu, Fe, I, and Se, respectively”. **should be replaced with** “AACM-100 or AACM-EZ-100: 60, 70, 8, 40, 1.0, and 0.250 mg kg^−1^ of Zn, Mn, Cu, Fe, I, and Se, respectively; AACM-70 or AACM-EZ70: 42, 49, 6, 28, 0.700, and 0.175 mg kg^−1^ of Zn, Mn, Cu, Fe, I, and Se, respectively; AACM-40 or AACM-EZ-40: 24, 28, 3, 16, 0.400, and 0.100 mg kg^−1^ of Zn, Mn, Cu, Fe, I, and Se, respectively”.

Table 6

**In the original publication, there was an error for** “AACM-100 or AACM-EZ-100: 60, 70, 8, 40, 0.250 mg kg^−1^ of Zn, Mn, Cu, Fe, I, and Se, respectively; AACM-70 or AACM-EZ-70: 42, 49, 6, 28, 0.175 mg kg^−1^ of Zn, Mn, Cu, Fe, I, and Se, respectively; AACM-40 or AACM-EZ-40: 24, 28, 3, 16, 0.100 mg kg^−1^ of Zn, Mn, Cu, Fe, I, and Se, respectively”. **should be replaced with** “AACM-100 or AACM-EZ-100: 60, 70, 8, 40, 1.0, and 0.250 mg kg^−1^ of Zn, Mn, Cu, Fe, I, and Se, respectively; AACM-70 or AACM-EZ70: 42, 49, 6, 28, 0.700, and 0.175 mg kg^−1^ of Zn, Mn, Cu, Fe, I, and Se, respectively; AACM-40 or AACM-EZ-40: 24, 28, 3, 16, 0.400, and 0.100 mg kg^−1^ of Zn, Mn, Cu, Fe, I, and Se, respectively”.

Table 7

**In the original publication, there was a mistake in the legend for** “AACM-100 or AACM-EZ-100: 60, 70, 8, 40, 0.250 mg kg^−1^ of Zn, Mn, Cu, Fe, I, and Se, respectively; AACM-70 or AACM-EZ-70: 42, 49, 6, 28, 0.175 mg kg^−1^ of Zn, Mn, Cu, Fe, I, and Se, respectively; AACM-40 or AACM-EZ-40: 24, 28, 3, 16, 0.100 mg kg^−1^ of Zn, Mn, Cu, Fe, I, and Se, respectively. mg cm^3^”. **should be replaced with** “AACM-100 or AACM-EZ-100: 60, 70, 8, 40, 1.0, and 0.250 mg kg^−1^ of Zn, Mn, Cu, Fe, I, and Se, respectively; AACM-70 or AACM-EZ70: 42, 49, 6, 28, 0.700, and 0.175 mg kg^−1^ of Zn, Mn, Cu, Fe, I, and Se, respectively; AACM-40 or AACM-EZ-40: 24, 28, 3, 16, 0.400, and 0.100 mg kg^−1^ of Zn, Mn, Cu, Fe, I, and Se, respectively. Mg/cm^3^”. 

Figure 1

**There was an error in the original publication for legends** “IM or IM-EZ: 60, 70, 8, 40, 0.250 mg kg^−1^ of Zn, Mn, Cu, Fe, I, and Se, respectively; AACM100 or AACM-EZ100: 60, 70, 8, 40, 0.250 mg kg^−1^ of Zn, Mn, Cu, Fe, I, and Se, respectively; AACM70 or AACM-EZ70: 42, 49, 6, 28, 0.175 mg kg^−1^ of Zn, Mn, Cu, Fe, I, and Se, respectively; AACM40 or AACM-EZ40: 24, 28, 3, 16, 0.10 mg kg^−1^ of Zn, Mn, Cu, Fe, I, and Se, respectively”. **should be replaced with** “IM or IM-EZ: 60, 70, 8, 40, 1.0, and 0.250 mg kg^−1^ of Zn, Mn, Cu, Fe, I, and Se, respectively; AACM100 or AACM-EZ100: 60, 70, 8, 40, 1.0, and 0.250 mg kg^−1^ of Zn, Mn, Cu, Fe, I, and Se, respectively; AACM70 or AACM-EZ70: 42, 49, 6, 28, 0.700, and 0.175 mg kg^−1^ of Zn, Mn, Cu, Fe, I, and Se, respectively; AACM40 or AACM-EZ40: 24, 28, 3, 16, 0.400, and 0.10 mg kg^−1^ of Zn, Mn, Cu, Fe, I, and Se, respectively”.

Figure 2

**There was an error in the original publication for legend** “IM or IM-EZ: 60, 70, 8, 40, 0.250 mg kg^−1^ of Zn, Mn, Cu, Fe, I, and Se, respectively; AACM100 or AACM-EZ100: 60, 70, 8, 40, 0.250 mg kg^−1^ of Zn, Mn, Cu, Fe, I, and Se, respectively; AACM70 or AACM-EZ70: 42, 49, 6, 28, 0.175 mg kg^−1^ of Zn, Mn, Cu, Fe, I, and Se, respectively; AACM40 or AACM-EZ40: 24, 28, 3, 16, 0.100 mg kg^−1^ of Zn, Mn, Cu, Fe, I, and Se, respectively. Data were analyzed by Dunnett’s test (*p* < 0.05); ° Differs from IM treatment; * Differs from IM-EZ treatment”. **should be replaced with** “IM or IM-EZ: 60, 70, 8, 40, 1.0, and 0.250 mg kg^−1^ of Zn, Mn, Cu, Fe, I, and Se, respectively; AACM100 or AACM-EZ100: 60, 70, 8, 40, 1.0, and 0.250 mg kg^−1^ of Zn, Mn, Cu, Fe, I, and Se, respectively; AACM70 or AACM-EZ70: 42, 49, 6, 28, 0.700, and 0.175 mg kg^−1^ of Zn, Mn, Cu, Fe, I, and Se, respectively; AACM40 or AACM-EZ40: 24, 28, 3, 16, 0.400, and 0.10 mg kg^−1^ of Zn, Mn, Cu, Fe, I, and Se, respectively”.

Figure 3

**There was a mistake in the original publication for** “IM or IM-EZ: 60, 70, 8, 40, 0.250 mg kg^−1^ of Zn, Mn, Cu, Fe, I, and Se, respectively; AACM100 or AACM-EZ100: 60, 70, 8, 40, 0.250 mg kg^−1^ of Zn, Mn, Cu, Fe, I, and Se, respectively; AACM70 or AACM-EZ70: 42, 49, 6, 28, 0.175 mg kg^−1^ of Zn, Mn, Cu, Fe, I, and Se, respectively; AACM40 or AACM-EZ40: 24, 28, 3, 16, 0.100 mg kg^−1^ of Zn, Mn, Cu, Fe, I, and Se, respectively”. **should be replaced with** “IM or IM-EZ: 60, 70, 8, 40, 1.0, and 0.250 mg kg^−1^ of Zn, Mn, Cu, Fe, I, and Se, respectively; AACM100 or AACM-EZ100: 60, 70, 8, 40, 1.0, and 0.250 mg kg^−1^ of Zn, Mn, Cu, Fe, I, and Se, respectively; AACM70 or AACM-EZ70: 42, 49, 6, 28, 0.700, and 0.175 mg kg^−1^ of Zn, Mn, Cu, Fe, I, and Se, respectively; AACM40 or AACM-EZ40: 24, 28, 3, 16, 0.400, and 0.10 mg kg^−1^ of Zn, Mn, Cu, Fe, I, and Se, respectively”.

Figure 4

**There was a mistake in the original publication for** “IM or IM-EZ: 60, 70, 8, 40, 0.250 mg kg^−1^ of Zn, Mn, Cu, Fe, I, and Se, respectively; AACM100 or AACM-EZ100: 60, 70, 8, 40, 0.250 mg kg^−1^ of Zn, Mn, Cu, Fe, I, and Se, respectively; AACM70 or AACM-EZ70: 42, 49, 6, 28, 0.175 mg kg^−1^ of Zn, Mn, Cu, Fe, I, and Se, respectively; AACM40 or AACM-EZ40: 24, 28, 3, 16, 0.100 mg kg^−1^ of Zn, Mn, Cu, Fe, I, and Se, respectively”. **should be replaced with** “IM or IM-EZ: 60, 70, 8, 40, 1.0, and 0.250 mg kg^−1^ of Zn, Mn, Cu, Fe, I, and Se, respectively; AACM100 or AACM-EZ100: 60, 70, 8, 40, 1.0, and 0.250 mg kg^−1^ of Zn, Mn, Cu, Fe, I, and Se, respectively; AACM70 or AACM-EZ70: 42, 49, 6, 28, 0.700, and 0.175 mg kg^−1^ of Zn, Mn, Cu, Fe, I, and Se, respectively; AACM40 or AACM-EZ40: 24, 28, 3, 16, 0.400, and 0.10 mg kg^−1^ of Zn, Mn, Cu, Fe, I, and Se, respectively”.

Figure 5

**There was a mistake in the original publication for** “IM or IM-EZ: 60, 70, 8, 40, 0.250 mg kg^−1^ of Zn, Mn, Cu, Fe, I, and Se, respectively; AACM100 or AACM-EZ100: 60, 70, 8, 40, 0.250 mg kg^−1^ of Zn, Mn, Cu, Fe, I, and Se, respectively; AACM70 or AACM-EZ70: 42, 49, 6, 28, 0.175 mg kg^−1^ of Zn, Mn, Cu, Fe, I, and Se, respectively; AACM40 or AACM-EZ40: 24, 28, 3, 16, 0.100 mg kg^−1^ of Zn, Mn, Cu, Fe, I, and Se, respectively”. **should be replaced with** “IM or IM-EZ: 60, 70, 8, 40, 1.0, and 0.250 mg kg^−1^ of Zn, Mn, Cu, Fe, I, and Se, respectively; AACM100 or AACM-EZ100: 60, 70, 8, 40, 1.0, and 0.250 mg kg^−1^ of Zn, Mn, Cu, Fe, I, and Se, respectively; AACM70 or AACM-EZ70: 42, 49, 6, 28, 0.700, and 0.175 mg kg^−1^ of Zn, Mn, Cu, Fe, I, and Se, respectively; AACM40 or AACM-EZ40: 24, 28, 3, 16, 0.400, and 0.10 mg kg^−1^ of Zn, Mn, Cu, Fe, I, and Se, respectively”.

## Text Correction

In Abstract, **there was an error in the original publication.** “The main effects included EZ supplementation (10,000 FTU kg^−1^) and AACM inclusion level (100, 70, and 40% of inorganic mineral recommendations), plus 2 control treatments”. **should be replaced with** “The main effects included EZ supplementation (600 FTU kg^−1^) and AACM inclusion level (100%, 70%, and 40% of inorganic mineral recommendations), plus two control treatments”.

In Section 2.2, **there was a mistake in the text for** “A total of 512 Dekalb White laying hens at 67 weeks of age were distributed into 64 experimental cages equipped with trough-type feeders and automatic drinkers with attached cups”. **should be replaced with** “A total of 512 Dekalb White laying hens at 68 weeks of age were distributed into 64 experimental cages equipped with trough-type feeders and automatic drinkers with attached cups”.

In the same section, **there was a mistake in the text for** “The experimental period consisted of 14 days of adaptation plus 5 cycles of 28 days each, totaling 154 days. During this period, water was available *ad libitum*”. **should be replaced with** “The experimental period consisted of 5 cycles of 28 days each, totaling 140 days. During this period, water was available *ad libitum*”.

In Section 2.3, **there was a mistake in the text for** “The first factor referred to diets with amino acid-complexed minerals (Zn, Mn, Cu, Fe, I, and Se), without (AACM) or with (AACM-EZ) the addition of 10,000 FTU kg^−1^ phytase”. **should be replaced with** “The first factor referred to diets with amino acid-complexed minerals (Zn, Mn, Cu, Fe, I, and Se), without (AACM) or with (AACM-EZ) the addition of 600 FTU kg^−1^ phytase”.

**There was a mistake in the text for** “The second factor corresponded to three levels of AACM inclusion (100, 70, or 40% AACM). Inclusion levels were based on the IM requirements of the Dekalb White manual guideline, which are 60, 70, 8, 40, and 0.250 of Zn, Mn, Cu, Fe, I, and Se, respectively (Table 1)”. **should be replaced with** “The second factor corresponded to 3 levels of AACM inclusion (100%, 70%, or 40% AACM). Inclusion levels were based on the IM requirements of the Dekalb White manual guideline, which are 60, 70, 8, 40, 1.0, and 0.250 mg kg^−1^ of Zn, Mn, Cu, Fe, I, and Se, respectively (Table 1)”.

In the same section, **there was a mistake in the text for** “Inclusion levels were based on the IM requirements of the Dekalb White manual guideline, which are 60, 70, 8, 40, and 0.250 of Zn, Mn, Cu, Fe, I, and Se, respectively (Table 1). Two control treatments were used, which corresponded to IM supplementation with (IM-EZ) or without 10,000 FTU kg^−1^ phytase”. **should be replaced with** “Inclusion levels were based on the IM requirements of the Dekalb White manual guideline, which are 60, 70, 8, 40, 1.0, and 0.250 mg kg^−1^ of Zn, Mn, Cu, Fe, I, and Se, respectively (Table 1). Two control treatments were used, which corresponded to IM supplementation with (IM-EZ) or without 600 FTU kg^−1^ phytase”.

**There was a mistake in the text for** “Experimental treatments included two basal diets (with and without the addition of phytase), where mineral premix was modified to create each treatment group”. **should be replaced with** “Experimental treatments included 2 basal diets (with and without the addition of phytase), where mineral premix was modified to create each treatment group”.

In Section 2.8.2, **there was a mistake for** “At the end of the experimental period, one bird was selected per experimental plot according to the average weight of each plot and euthanized by cervical dislocation to collect tibia”. **should be replaced with** “At the end of the experimental period, 1 bird was selected per experimental plot according to the average weight of each plot and euthanized by cervical dislocation to collect tibia”.

In Section 2.9, **there was a mistake for** “Subsequently, the images were analyzed using Dicom software (version 1.1.7, Horos, Purview, Annapolis, MD, USA) to estimate the individual values of bone radiodensity at three different diaphysis section levels (proximal, medial, and distal). Each region was divided into four quadrants, and a circular region of interest was selected for densitometric evaluation of the cortical bone”. **should be replaced with** “Subsequently, the images were analyzed using Dicom software (version 1.1.7, Horos, Purview, Annapolis, MD, USA) to estimate the individual values of bone radiodensity at 3 different diaphysis section levels (proximal, medial, and distal). Each region was divided into 4 quadrants, and a circular region of interest was selected for densitometric evaluation of the cortical bone”.

In Section 3.6, **there was a mistake for** “There was an interaction between diets and AACM levels on bone density in the three studied regions: proximal (*p* = 0.02), medial (*p* = 0.01), and distal (*p* = 0.04)”. **should be replaced with** “There was an interaction between diets and AACM levels on bone density in the 3 studied regions: proximal (*p* = 0.02), medial (*p* = 0.01), and distal (*p* = 0.04)”.

In Section 4, **there was a mistake for** “Phytase’s ability to enhance phosphorus and mineral availability may have influenced the utilization of AACM components differently at this specific concentration, modulating metabolic processes and influencing EO [26]”. **should be replaced with** “Phytase’s ability to enhance P and mineral availability may have influenced the utilization of AACM components differently at this specific concentration, modulating metabolic processes and influencing EO [26]”.

**There was a mistake for** “Eggshell quality is a crucial aspect of the poultry industry’s economic viability, as losses due to egg breakage can account for 8 to 10% of total production [36]”. **should be replaced with** “Eggshell quality is a crucial aspect of the poultry industry’s economic viability, as losses due to egg breakage can account for 8% to 10% of total production [36]”.

**There was a mistake for** “Phytic acid, present in feedstuffs under natural conditions, has a high potential for complexation with positively charged molecules, such as Fe+3 and Cu+2 cations, forming insoluble complexes with phytate when in the ionic form [53,54]”. **should be replaced with** “As part of this research, the use of phytase resulted in an increase in the concentration of red blood cells. Phytic acid, present in feedstuffs under natural conditions, has a high potential for complexation with positively charged molecules, such as Fe^+3^ and Cu^+2^ cations, forming insoluble complexes with phytate when in the ionic form [53,54]”.

The authors state that the scientific conclusions are unaffected. This correction was approved by the Academic Editor. The original publication has also been updated.
